# Transitional circulation and hemodynamic monitoring in newborn infants

**DOI:** 10.1038/s41390-022-02427-8

**Published:** 2023-01-02

**Authors:** Aravanan Anbu Chakkarapani, Charles C. Roehr, Stuart B. Hooper, Arjan B. te Pas, Samir Gupta, On behalf of the ESPR Neonatal Resuscitation section writing group

**Affiliations:** 1 Division of Neonatology, Sidra Medicine, Doha, Qatar; 2 Weill Cornell Medicine, Doha, Qatar; 3 National Perinatal Epidemiology Unit, Nuffield Department of Population Health, Medical Sciences Division, University of Oxford, Oxford, UK; 4 Newborn Services, Southmead Hospital, North Bristol Trust, Bristol, UK; 5 Faculty of Health Sciences, University of Bristol, Bristol, UK; 6 Department of Obstetrics and Gynaecology, Monash University, Melbourne, VIC Australia; 7 The Ritchie Centre, Hudson Institute for Medical Research, Melbourne, VIC Australia; 8 Neonatology, Willem Alexander Children’s Hospital, Leiden University Medical Center Leiden, Leiden, The Netherlands; 9 Durham University, Durham, UK

## Abstract

**Abstract:**

Transitional circulation is normally transient after birth but can vary markedly between infants. It is actually in a state of transition between fetal (in utero) and neonatal (postnatal) circulation. In the absence of definitive clinical trials, information from applied physiological studies can be used to facilitate clinical decision making in the presence of hemodynamic compromise. This review summarizes the peculiar physiological features of the circulation as it transitions from one phenotype into another in term and preterm infants. The common causes of hemodynamic compromise during transition, intact umbilical cord resuscitation, and advanced hemodynamic monitoring are discussed.

**Impact:**

- Transitional circulation can vary markedly between infants.
- There are alterations in preload, contractility, and afterload during the transition of circulation after birth in term and preterm infants.
- Hemodynamic monitoring tools and technology during neonatal transition and utilization of bedside echocardiography during the neonatal transition are increasingly recognized.
- Understanding the cardiovascular physiology of transition can help clinicians in making better decisions while managing infants with hemodynamic compromise.
- The objective assessment of cardio-respiratory transition and understanding of physiology in normal and disease states have the potential of improving short- and long-term health outcomes.

## Introduction

Understanding the physiological changes that occur during the transition to newborn life is essential to correctly interpret the hemodynamic issues that may occur during and after this process. It is challenging for neonatologists to manage circulatory failure during the transition;^1^ issues can differ between extreme preterm infants and term infants because premature infants have an immature circulation,^2^ whereas circulatory systems can be malformed in term infants. Hence, the approach should be adopted for specific pathophysiological conditions such as patent ductus arteriosus (PDA), hypotension, intraventricular hemorrhage, birth asphyxia, severe growth restriction and pulmonary hypertension as the circulation transitions.^3–5^

This review summarizes the distinct features of transitional circulation and intact umbilical cord resuscitation during the transition. Some newer concepts of hemodynamic monitoring (neonatologist performing echocardiography, near-infrared spectroscopy (NIRS), electrical velocimetry) during the transition are also discussed that are being increasingly utilized at the bedside.

## Physiology of fetal circulation

In the fetus, blood oxygenation occurs in the placenta as the fetal lungs are filled with liquid and do not function as an organ of gas exchange.^6,7^ In humans, the single umbilical vein carries oxygenated blood from the placenta to the left atrium via the ductus venosus, which joins the IVC close to the IVC-right atrial junction. At the same time, deoxygenated blood from the lower part of the body flows via the IVC into the right atrium. Interestingly, these two (oxygenated and deoxygenated blood) flows do not mix due to the shape of the ductus venosus and the presence of the ridge of Eustachian valve. Therefore, most of the oxygenated blood flows toward the foramen ovale and enters the left atrium and reaches the left ventricle. As a result, in the fetus, the left ventricle receives its preload primarily from the organ of gas exchange (placenta) just like the adult (lung). Deoxygenated blood from IVC mixes with the SVC flow and enters the right ventricle.^4,8,9^

In the fetus, the right ventricle pumps deoxygenated blood into the main pulmonary artery during systole. However, because pulmonary vascular resistance (PVR) is so high, the majority (~90%) of right ventricular (RV) output bypasses the lungs and enters the systemic circulation via DA. As systemic vascular resistance (SVR) is lower than PVR, due to the low-resistance placental bed, this also contributes to the high rate of “right-to-left” shunting through the DA. In the post-ductal descending aorta, blood flow is derived from both the left and right ventricle (via DA) and flows toward the lower body, including the placenta where it gets oxygenated.^3,4,10^ As the right ventricle provides the majority (>90%) of this flow, it provides the majority of flow to the organ of gas exchange (placenta) just like the adult. In contrast, the left ventricle provides the vast majority of flow to the upper body, with only ~10% of left ventricular (LV) output mixing with RV output and flowing to the lower body. As a result, blood in preductal arteries (arteries arising from the aorta prior to the junction with the DA) is more highly oxygenated.

## Physiology of transitional circulation

**Fig. 1:**
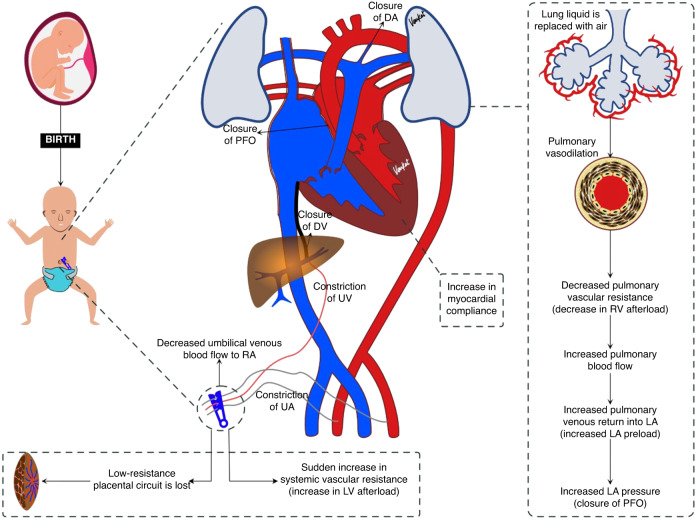
Features of transitional circulation. After cord clamping the systemic vascular resistance increases, lungs aerate and fetal shunts close functionally; the series of changes depicted in the figure allow transition from fetal to neonatal circulation.

The transitional circulation (Fig. 1) refers to the period of time when fetal circulation transforms into the neonatal phenotype.^11,12^ Soon after birth, this phase starts when the umbilical cord is clamped, and the lungs aerate following the first few breaths. The physiological adaptation following the delivery of a newborn is a complex process that requires numerous adaptive changes to happen simultaneously in various organs. The most significant being the respiratory and cardiovascular systems. Immediately after birth, two outcomes influence whether the newborn will transition successfully or not.^4,10^

Firstly, lung liquid is replaced by air with the onset of air breathing.^13^ While the mechanism is unclear, the movement of air into the lung is a potent pulmonary vasodilator. Lang et al.’s study demonstrated that a vagal reflex may mediate a rapid global increase in pulmonary blood flow (PBF) in response to partial lung aeration.^14^ This causes a large decrease PVR and a large and rapid increase (~30 fold) in PBF. As PVR rapidly falls below SVR, shunting across the DA reverses (becomes left-to-right) allowing LV output to contribute to PBF.^15,16^ Through this process, more deoxygenated blood is carried to the lung for oxygenation. Pulmonary veins drain the oxygenated blood from the lungs into the left atrium, which then increases the lungs' contribution to LV preload,^3,15^ with the lungs eventually becoming the sole source of this preload.

Secondly, with umbilical cord clamping, umbilical venous blood flow via ductus venosus and foramen ovale decreases and so preload entering the left ventricle via the left atrium decreases. Clamping the umbilical artery also removes the low-resistance placenta circuit exposing the neonatal left ventricle to a sudden increase in SVR (increase in LV afterload), causing a sudden “step-like” increase in arterial blood pressure and blood flow to the upper body.^4,17^

To understand the changes that happen during the transition, one must first have knowledge of cardiovascular structure and function in the fetus.

## Fetal shunts during transitional circulation

Soon after birth, all three fetal shunts go through a series of changes as detailed below.^3,18,19^

### Ductus venous

Following clamping of the umbilical cord, the ductus venosus becomes operationally ineffective due to decreased umbilical venous blood flow. Ductus venosus closes permanently within the first 2 weeks after birth, although closure is slower in preterm infants than in term infants.^4,18,19^

### Foramen ovale

In utero, right atrial pressure is higher than left atrial pressure. Because of this, the foramen ovale remains open. Following lung aeration, the decrease in PVR increases pulmonary artery flow, which in turn increases pulmonary venous return to the left atrium. The resulting increase in left atrial pressure combined with the loss of directed flow from the ductus venosus, the foramen ovale closes and fuses with the interatrial septal wall. This process makes the foramen ovale operationally ineffective soon after birth.^20^ Foramen ovale permanently closes within the first few years of life.^4,18,19^

### Ductus arteriosus (DA)

The majority (~90%) of RV output is shunted from the pulmonary artery to the descending Aorta (known as right-to-left shunting) through the DA, with flow continuing throughout the cardiac cycle. The flow during diastole results from retrograde PBF exiting the pulmonary circulation and traversing the DA due to the closure of the RV valve and the low SVR. Following lung aeration, PVR rapidly decreases below SVR, particularly after umbilical cord clamping, causing the shunt across the DA to become bidirectional. The flow is mostly left to right but is initially right-to-left during early systole because the flow wave exiting the right ventricle reaches the pulmonary artery end of the DA before the flow wave exiting the left ventricle reaches the aortic end. When flow emanating from the left ventricle reaches the aortic end of the DA, DA flow reverses and becomes left-to-right during late systole and throughout diastole. While this beat-to-beat flow reversal likely contributes DA closure, increased oxygenation, and a reduction in circulating prostaglandin levels facilitates the contraction of the smooth muscle and eventually closure of the DA. PVR may not fall in the usual fashion in premature infants due to lung immaturity and in term infants with lung hypoplasia. Hence, the closure of the DA varies depending upon underlying pathophysiology. It becomes ligamentum arteriosum in the later part of life.^4,18,19^

## Transitional circulation in term newborns

In the majority of term infants, the transition to ex utero life happens smoothly. We discuss below some alterations in preload, contractility, and afterload during term during transition of the circulation after birth.^3,21^

### Preload changes during transition

The effect of umbilical cord clamping on preload to the left and right ventricles depends on whether the lungs have aerated. When cord clamping occurs before lung aeration and the increased PBF, the sudden loss in venous return to the left and right ventricles causes a reduction in cardiac output that is only restored after the lungs aerate and PBF increases. This explains the low heart rates seen in nomograms (50% of infants <100 bpm at 1 min) of healthy infants at birth following immediate cord clamping.^22^ In contrast, lung aeration prior to cord clamping avoids this reduction in heart rate as the increase in PBF replaces the loss in preload caused by cord clamping.^23,24^

The myocardium of neonates is much less compliant than that of adults, leading to partial diastolic dysfunction after birth. As a result, passive filling of ventricles from atria (E wave in Doppler) is lower than active atrial contraction (A wave) mediated ventricular filling.^25^

### Myocardial contractility changes during transition

Neonates have less myocardial mass than adults and the organization of myocardial muscle is different in neonates, which gradually matures over time. Cardiac output is determined by venous return, heart rate and stroke volume.^16^ Noori et al. used echocardiography during the transitional circulation period and observed a significant (*P* < 0.001) decline in heart rate (169 ± 20 beats/min at 3–7 min, 153 ± 17 beats/min at 9–14 min, 148 ± 16 beats/min at 15–19 min) after birth. On the contrary, a significant (*P* < 0.001) increase in LV stroke volume (1.01 ± 0.23 ml/kg/min at 3–7 min, 1.23 ± 0.22 ml/kg/min at 9–14 min, 1.28 ± 0.21 ml/kg/min at 15–19 min) was observed after birth. However, LV output was non-significantly (*P* = 0.10) increased from 168 + 42 ml/kg/min at 3–7 min, 186 + 26 ml/kg/min at 9–14 min, 189 + 27 ml/kg/min at 15–19 min after birth. It is interesting to note that an increase in LV stroke volume is not likely due to changes in contractility. Increased preload is the predominant contributor.^16,26,27^

### Afterload changes during transition

The change in afterload during transition also depends on whether cord clamping occurs before or after lung aeration. When cord clamping occurs before lung aeration, the sudden loss of the low-resistance placental bed causes a stepwise, sudden (within 1 s) increase in SVR and arterial pressure of ~30%.^23^ However, when lung aeration begins before cord clamping, the large decrease in PVR contributes to an overall decrease in total body vascular resistance, leading to a reduction in afterload. As a result, clamping the umbilical cord only causes a small increase in arterial blood pressure, as the lungs become an alternate pathway (via the DA) for low-resistance blood flow for the LV. As the DA closes, LV takes has time to gradually adapt to the increase in afterload caused by the loss of the placental circulation. Although RV afterload decreases due to the decrease in PVR following lung aeration, pressures in the pulmonary artery remain high (slightly below aortic pressures) while the DA remains open.^21^ It is only when the DA closes and the two circulations functionally separate that pulmonary arterial pressures can substantially decrease. In term babies, FO closes immediately after birth because of the sudden increase in left atrial pressure (increased pulmonary venous return), and this has no clinical relevance.

Overall, the term neonatal myocardium functions according to the baby’s needs under normal transition. However, if the transition is compromised by abrupt changes in preload or afterload, the myocardium might not be able to manage the situation and eventually fail. Hence, understanding the status of the term neonatal myocardium and the pathophysiologic state of the baby (e.g., hypoxic-ischemic encephalopathy (HIE), pulmonary hypertension), can guide hemodynamic management decisions and interventions based on the correct physiological principles.

## Transitional circulation in preterm neonates

In preterm neonates, the transition to ex utero life is likely to be compromised by various factors.^3,15,28–30^

### Pulmonary vascular resistance

Most preterm infants are born with immature lungs, especially extremely low gestational age newborns (ELGANs), who develop moderate to severe respiratory distress syndrome. While lung aeration is a potent stimulus for a decline in PVR, just like in term infants, underdevelopment of the pulmonary vascular bed structurally limits the extent to which PVR can decrease. As a result, the right ventricle has to cope with increased afterload and the reduced pulmonary artery blood flow reduces LV preload and cardiac output.^30–32^

### Systemic vascular resistance

After birth, the left ventricle of preterm babies, like term babies, has to pump blood against increased afterload due to the loss of the low resistant placental circulation. However, immaturity of the myocardium renders the LV less capable of coping with the high afterload. Combined, the decrease in LV preload with an increase in LV afterload accentuates decreased LV cardiac output.^30,31^

### Myocardial contractility and compliance

In addition to the discussion under section “Systemic vascular resistance”, premature myocardium has higher water content and less contractile mass than term neonates. This predisposes to the risk of myocardial failure in certain situations like acute increase in preload or afterload.^30,31^

### Persistence of fetal shunts

Fetal shunts do not close in the usual time frame in premature neonates although the precise mechanisms are unclear. However, left-to-right shunting through the DA and FO is common in preterm neonates after birth. This left to right shunt occurs when PVR is lower than SVR. In babies with hemodynamically significant PDA, significant blood volume shunts from aorta to pulmonary artery through DA. This causes a decrease in blood flow into the descending aorta and manifests as absent or reversed end diastolic flow, which is commonly referred to as “ductal steal”. Nevertheless, patent foramen ovale and PDA play a key role in optimizing circulation and end-organ perfusion in specific pathophysiological conditions.^30,31^

## Hemodynamic monitoring during transitional circulation

### Echocardiography

**Fig. 2:**
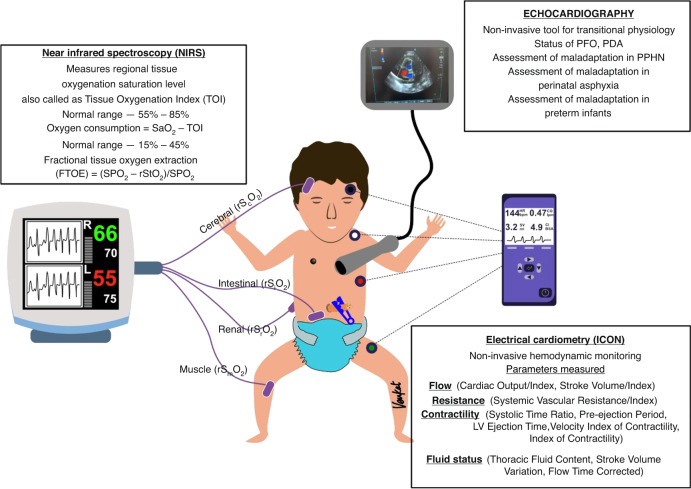
Hemodynamic (near-infrared spectroscopy (NIRS), electrical velocimetry (ICON), echocardiography) monitoring devices. The paramenters utilised for transitional circulation monitoring using NIRS, ICON and Echocardiography are described.

Echocardiography (Fig. 2) is an excellent non-invasive bedside tool to identify the cardiovascular changes during the transition from in utero to postnatal life.^33^ Normally, the cardiovascular adaptation that occurs during transition happens seamlessly without any hemodynamic compromise. However, the transition can be compromised during pathological states such as perinatal asphyxia, severe pulmonary hypoplasia, and prematurity. The key areas to concentrate on during bedside echocardiography to assess transition include the persistence of fetal shunts (PDA and patent foramen ovale), pulmonary pressures and cardiac function.^25,34,35^

#### Patent foramen ovale during transition

Soon after birth, bidirectional or left to right shunt across the foramen ovale is physiologically normal. However, an exclusive right to left (100%) shunt across the foramen ovale should always arouse suspicion of underlying complex congenital heart diseases such as tricuspid atresia or total anomalous pulmonary venous drainage.^34,35^

#### Patent ductus arteriosus (PDA) during transition

Like PFO shunting, bidirectional or left to right shunt across DA is a normal physiological event. However, following lung aeration, a persisting right to left shunt over 30% of cardiac cycle across the DA should prompt a detailed assessment to rule out persistent pulmonary hypertension (PPHN), duct-dependent systemic circulation, or duct-dependent pulmonary circulatory conditions.^34,35^

### Echocardiographic assessment of maladaptation in persistent pulmonary hypertension

The hallmarks of PPHN are raised pulmonary artery pressures caused by a persisting high PVR.^36^ These parameters are challenging to measure using bedside echocardiography^35^ and so an indirect approach for assessing the severity of pulmonary hypertension is commonly used.^37–39^ This involves bedside echocardiography^38^ that:

- Estimate the tricuspid regurgitation (an indirect measure of systolic pulmonary artery pressure (SPAP) or RV systolic pressure).
- Estimate the pulmonary regurgitation peak velocity (diastolic pulmonary artery pressure).
- Assess the shunt across PDA (in PPHN, the PDA shunt may be bidirectional or 100% right to left), transductal right to left flow peak velocity (SPAP).
- Estimate the LV systolic eccentricity index, assess the interventricular (IV) septum, and left ventricle shape: the IV septum might be flattened, D-shaped and banana-shaped LV in PPHN.
- Estimate the RV systolic time intervals (pulmonary artery acceleration time (PAAT), PAAT/RVET (RV ejection time) ratio).
- Recognize the RV hypertrophy with or without RV dilatation.
- Assessment of RV systolic function (tricuspid annular pan-systolic excursion).
- Estimate the RV fractional area change for RV systolic function.
- Measure LV and RV output (in PPHN, the cardiac output might be decreased due to LV and RV dysfunction).
- Assess the shunt across PFO: the PFO shunt might be bidirectional in PPHN.

### Echocardiographic assessment of maladaptation in perinatal asphyxia

Severe perinatal asphyxia results in a lack of oxygen and blood flow to the brain at birth, placing the infant at high risk of hypoxic-ischemic brain injury.^35^ As the severity of hypoxia deepens, myocardial function gradually declines. This decline counteracts the “brain saving” vasodilation of the cerebral circulation combined with vasoconstriction of the peripheral circulation, which is designed to increase blood flow and oxygen supply to the brain and other vital organs (e.g., myocardium).^40,41^ The peripheral vasoconstriction includes pulmonary circulation, which reduces the capacity of the lung to both exchange respiratory gases and supply venous return to the left ventricle. The hypoxia also inhibits breathing activity creating a vicious cycle that requires significant intervention This unavoidably involves IPPV to aerate the lungs and may also include chest compressions and adrenaline administration to enhance circulatory function. Then, during the rebound recovery phase, a maximally vasodilated cerebral circulation, exposes the delicate microvasculature to high pressures and flows, particularly if the autoregulatory protective mechanisms are dysfunctional.

Therapeutic hypothermia (TH) (reduction of core body temperature of 33.5 °C for 72 h) is the standard treatment for moderate to severe HIE in developed countries. TH should start within 6 h after birth based on specific criteria. In addition to ongoing hemodynamic compromise due to HIE, TH^42,43^ can cause cardiovascular complications in the following pathways.

- TH increases PVR and could lead to PPHN, which in turn leads to a rise in RV afterload and compromise in RV function.
- TH increases SVR due to peripheral vasoconstriction, leading to an increase in LV afterload and compromising LV function.
- TH causes bradycardia and hypotension, which leads to a decrease in cardiac output.

All of the mentioned pathways lead to decreased cardiac output and poor end-organ perfusion. TH has the potential to compromise cardiac function and hemodynamics and cause acidemia.

The goal of clinical care in infants with HIE and undergoing TH is to optimize end-organ blood flow. Bedside echocardiography is a valuable tool to monitor real-time hemodynamic changes following perinatal asphyxia. The echocardiographic assessment of the preload, cardiac function and afterload helps in clinical decision making in moderate to severe HIE patients with hemodynamic compromise. For example, in HIE infants undergoing TH, treatment can be objectively directed based on the underlying pathophysiology such as, whether to give normal saline bolus or not, selection of inotrope and monitoring of cardiac function and PVR for titrating treatment.

### Echocardiographic assessment of maladaptation in premature neonates

This has been discussed in detail under section “Hemodynamic monitoring during transitional circulation”^28^ and should focus on the following points:

- Assessment of physiologic fetal shunts and their hemodynamic significance.
- Assessment of biventricular function and correlation with perfusion.
- Assessment of pulmonary pressures in cases of severe respiratory distress syndrome and poorly oxygenating ELGAN.
- Assess response to medical treatment and interventions to guide management.
- Assess cardio-respiratory interaction to optimize support.

### Non-invasive cardiac output monitoring

In neonates, the gold standard for assessing of cardiac output is the intermittent pulmonary artery thermodilution technique or the Fick principle. However, both are invasive techniques and are not suitable for routine assessment in the neonatal unit.^44^

Non-invasive techniques are trans-thoracic echocardiography (TTE), ultrasound cardiac output monitor, and cardiac magnetic resonance imaging. These technologies are labor and skill intensive and only provide intermittent point assessment values, and not a continuous trend.^44^ TTE can be used to estimate ventricular outputs.

A recent systematic qualitative review^44^ on the accuracy and trending ability of electrical biosensing technology for non-invasive cardiac output monitoring (electrical velocimetry) (Fig. 2) in neonates has assessed 15 studies. They found that thoracic electrical biosensing technology (TEBT) gives reasonable accuracy, poor precision, and non-interchangeability compared with TTE, although a high degree of heterogeneity was noted between these studies, which affected the analyses. Thus, currently, TEBT should be used cautiously for monitoring and clinical decision making in neonates in the unit. However, new studies on TBET trend monitoring are needed to further evaluate its use in neonates during transitional circulation.

### Assessment of end-organ perfusion by using near-infrared spectroscopy (NIRS)

NIRS (Fig. 2) is a non-invasive tool to measure the difference in absorption of near-infrared light by oxy- and deoxyhaemoglobin to calculate regional tissue oxygen saturation (rS_t_O_2_) and fractional tissue oxygen extraction (FTOE).^45,46^ It is a frequently used technique in NICU and DR that gives continuous data on tissue oxygenation status in neonates, a surrogate of end-organ perfusion. Established reference values of rS_t_O_2_ and FTOE are available in the literature during transition for term and preterm infants.^47,48^ In a randomized controlled (*n* = 30) feasibility trial in infants <34 weeks, Pichler et al. reported that NIRS monitoring can reduce the burden of cerebral hypoxia during the transition and guide respiratory support and supplemental oxygen therapy.^49^

## Umbilical cord management strategies during transition

As indicated above, when the umbilical cord is clamped in the delivery room before the lung has aerated, LV preload and cardiac output decrease markedly. This is because LV preload in the fetus mainly depends upon umbilical venous return via the ductus venosus as pulmonary venous return is low before birth. As such, LV preload will remain low until the lung aerates and PBF increases. While this transition in circulatory physiology may appear complex, in practice it is straightforward for most well-term infants because they commence breathing and aerate their lungs immediately after birth. However, this hemodynamic transition is considerably more critical in preterm infants and in compromised term infants who have difficulty in aerating their lungs. As maintaining adequate LV preload is vital for sustaining an adequate LV cardiac output, initiating lung aeration to stimulate the increase in PBF prior to cord clamping is logical.^50^ This allows pulmonary venous return to immediately replace umbilical venous return as a source of LV preload following umbilical cord clamping.

Pulsation of the umbilical cord (or lack thereof) is often used to determine umbilical cord clamping time during delayed cord clamping, but it is a poor predictor of blood flow in the cord, with pulsations continuing well after flow has ceased.^51^ Blood flow in the umbilical cord continues for many minutes after birth (5–7 min),^51^ although this can vary considerably depending on the timing of uterotonic administration and the rate of increase in PBF. Uterotonic-induced contractions can cause umbilical flows to cease^52^ and the decrease in PVR redirects RV output toward the lungs and away from the placenta, causing a marked decrease in umbilical blood flow.^53^

A placental transfusion to the neonate can potentially occur for up to 5 min after delivery leading to the net movement of 28 ml/kg of blood from the placenta into the infant. However, this “placental transfusion” has yet to be explained scientifically as it is not gravity dependent^54^ and is not caused by uterine contractions,^52^ although the pressure changes caused by inspiration may play a role.^17^ Nevertheless, delayed umbilical cord clamping does result in increased hemoglobin stores and an increase in blood volume is the only feasible explanation for the increased birth weights of infants receiving delayed cord clamping at birth. However, as no antenatal measurements have ever been made, it is possible that labor causes a net shift of blood from the infant into the placenta (due to an increase in internal body pressures), which shifts back after birth to restore the balance.^55^ This would explain why placental transfusion is not evident in infants delivered by cesarean section.

**Fig. 3:**
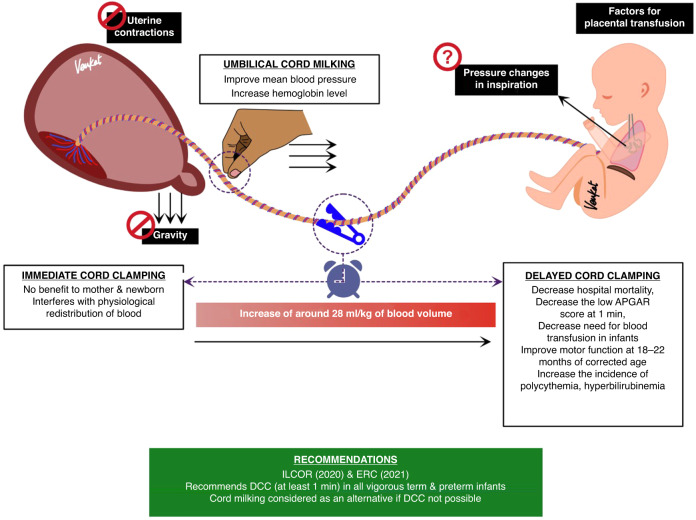
Cord management strategies. The limitaions of immediate cord clamping and benefits of delayed cord clamping & cord milking are described.

In 2021, Jasani et al.^56^ assessed the effect of four different umbilical cord management strategies (Fig. 3) for preterm infants by conducting a systematic review, and network meta-analysis from 6852 preterm infants enrolled in 56 studies.

- Immediate umbilical cord clamping (ICC).
- Delayed umbilical cord clamping (DCC).
- Umbilical cord milking (UCM).
- UCM and DCC.

This analysis showed that DCC was associated with the lower odds of mortality in preterm infants compared with ICC. DCC and UCM were related to reductions in intraventricular hemorrhage and the need for packed red cell transfusion compared with ICC. No significant difference was found between UCM and DCC for any outcome in infants over 29 weeks’ gestation. However, more recently, UCM in extremely preterm infants was found to increase the risk of severe IVH 4-fold and as such is not recommended for preterm infants <28 weeks.^57^

## International consensus on umbilical cord management

- The International Liaison Committee on Resuscitation (ILCOR) 2020 Consensus on Science and Treatment Recommendations (CoSTR) for Neonatal Life Support guideline advocate DCC (for at least 1 min) in uncompromised term and preterm infants.^58,59^
- The European Resuscitation Council (ERC) 2021 Guideline 2021 on Newborn resuscitation and support of transition of infants at birth recommends^60^ delayed cord clamping (DCC) for at least 60 s, ideally after the lungs are aerated. Cord milking should be considered as a reasonable alternative if DCC is not possible in infants more than 28 weeks’ gestation.

## Umbilical cord management in congenital diaphragmatic hernia (CDH)

The literature regarding the physiological adaptation of babies with CDH during transition are scarce,^61^ but some scientific studies are emerging. Lung aeration does not happen quickly in newborns with a DH because the lungs are small and the intrathoracic space-occupying effects of herniated abdominal organs. Three components of CDH are (1) pulmonary hypoplasia, (2) pulmonary hypertension, and (3) cardiac dysfunction.

Apart from a small lung with a limited gas exchange surface area, CDH infants also have an underdevelopment pulmonary vascular bed with a small cross-sectional area. As such, CDH infants face significant hemodynamic challenges during the transition and are at high risk of developing pulmonary hypertension and cardiac dysfunction (LV dysfunction, RV dysfunction or biventricular dysfunction).

The standard delivery room approach is to do immediate cord clamping followed by intubation on the resuscitation table. However, studies in lambs with a DH and severe lung hypoplasia have shown that ventilating them for up to 10 min “on the cord” (umbilical cord intact) results in markedly lower PVR and higher PBF for up to 2 h after birth compared with lambs receiving immediate cord clamping. Foglia et al.^62^ did a single-site pilot (20 babies), safety and feasibility study of initiating resuscitation by intubation and ventilation before umbilical cord clamping in >36-week gestational age babies with CDH with the help of a trolley. The study team could place 100% of babies on the trolley for resuscitation and intubate 85% of babies before UCC. Currently, a high-quality multi-center randomized controlled trial is underway examining the benefits of PBCC, which involves ventilating infants with the umbilical cord intact.

## Devices used for resuscitation with an intact umbilical cord to establish ventilation during transition circulation in the delivery room

The authors of ILCOR and ERC presently advise cutting the umbilical cord and initiating standard resuscitative measures in infants who are not breathing. Intact umbilical cord resuscitation to establish ventilation prior to cord clamping for compromised babies at birth is currently on trial and hence not yet recommended for routine use.^50,63–65^

To address this issue in detail, we need a platform that supports the initiation of resuscitation (establishing ventilation) with an intact umbilical cord while the fetoplacental circulation remains intact. This platform should accommodate essential equipment required for resuscitation, be stable so that the required actions can be undertaken, provide an external heat source for the infant and be able to be placed close to the mother so that the umbilical cord is not stretched. We need more studies to assess the benefits of this approach in high-risk preterm deliveries which should provide more information to clinicians about the potential benefits. However, in using these devices, consideration should be given as to how the platform will be positioned in the delivery room and the position of care providers (obstetricians, neonatologists, anesthesiologists, and nurses) around the platform during resuscitation in vaginal and cesarean section deliveries. This may include the “scrubbing in” of neonatologists and the sterilization of resuscitation equipment during cesarean section deliveries to maintain a sterile field while the infant is being stabilized or resuscitated on the cord, as occurred in the ABC and BabyDUCC trials.^24,66^

## Conclusion

After birth, significant cardiopulmonary changes occur gradually in a sequential manner, changing from the fetal circulatory phenotype, through to a transitional circulation phenotype, to finally the definitive neonatal/ adult circulation. During the transition, the key areas to focus on for the circulation are maintaining preload following cord clamping, cardiac contractility (systolic function), minimizing the increase in afterload, increasing myocardial compliance (diastolic function), and minimizing the persistence of fetal shunts. In addition, studies assessing end-organ perfusion and cerebral oximetry during the neonatal transition are urgently required. Immaturity of the circulatory system in preterm infants enhances hemodynamic compromise during the transition. We need more physiological studies with advanced hemodynamic monitoring technologies to understand the changes that happen during the transition and during ventilation with intact umbilical cord resuscitation to improve the outcomes for neonates during this crucial period. Understanding the cardiovascular physiology of transition can help clinicians make better decisions while managing infants with hemodynamic compromise.
